# Body Mass Index Screening and Follow-Up: A Cross-Sectional Questionnaire Study of Pennsylvania School Nurses

**DOI:** 10.2196/11619

**Published:** 2018-12-21

**Authors:** Erica Francis, Alicia Marie Hoke, Jennifer Lynn Kraschnewski

**Affiliations:** 1 Department of Pediatrics Penn State University Hershey, PA United States

**Keywords:** body mass index, childhood overweight, childhood obesity, pediatric obesity, Pennsylvania, school nursing, Web-based survey

## Abstract

**Background:**

Childhood overweight and obesity health concerns can affect a student’s academic performance, so it is important to identify resources for school nurses that would help to improve self-efficacy, knowledge, and confidence when approaching parents with sensitive weight-related information and influence overall obesity prevention efforts in the school setting.

**Objective:**

The purpose of this study was to conduct a Pennsylvania (PA) state-wide 29-item survey addressing school nursing barriers and practices, supplementing information already known in this area. Although the survey covered a range of topics, the focus was body mass index (BMI) screening and its related practice within the schools.

**Methods:**

We conducted a state-wide Web-based survey of school nurses in PA to understand current areas of care, find ways to address child health through school BMI screenings and follow up, and identify current educational gaps to assist school nurses with providing whole child care within the realm of weight management. Chi-square test of independence was conducted to determine the relationship between BMI screening follow up and interest in a BMI toolkit.

**Results:**

Nurse participants (N=210), with a 42% (210/500) response rate, represented 208 school districts across PA. Participants were asked about their current process for notifying parents of BMI screening results. The majority (116/210, 55.2%) send a letter home in the mail, while others (62/210, 29.5%) send a letter home with students. A small number (8/210, 3.8%) said they did not notify parents altogether, and some (39/210, 18.6%) notify parents electronically. More than one-third (75/210, 35.7%) of nurses reported receiving BMI screening inquiries from parents; however, under half (35/75, 46.7%) of those respondents indicated they follow up with parents whose child screens overweight or obese. Overall, the vast majority (182/210, 86.7%) do not follow up with parents whose child screens overweight or obese. The majority (150/210, 71.4%) of the nurses responded they would benefit from a toolkit with resources to assist with communication with parents and children about BMI screenings. A significant association between respondent follow up and interest in a BMI toolkit was observed (*P*=.01).

**Conclusions:**

Schools must start recognizing the role school nurses play to monitor and promote children’s health. This goal might include involving them in school-based preventive programs, empowering them to lead initiatives that support whole child health and ensuring opportunities for professional development of interest to them. Nonetheless, the first step in facilitating obesity prevention methods within schools is to provide school nurses with meaningful tools that help facilitate conversations with parents, guardians, and caregivers regarding their child’s weight status and health through a BMI screening toolkit.

## Introduction

Rising rates of childhood overweight and obesity contribute to increased risk of chronic disease and other health concerns [1,2] that directly affect a student’s academic performance [3]. While many environmental factors play a role in the rising prevalence of obesity, school-based health services hold enormous potential to affect children’s health outcomes given the fact that children spend the majority of their waking hours in school settings [4]. Unfortunately, the School Health Policies and Practices Study [5] indicates few services linked to obesity prevention despite school nurses’ ability and interest to champion strategies to curb rising obesity rates [6]. School nurses are limited in the knowledge and resources available for addressing the topic of childhood obesity prevention [7]. As a result, there is an inconsistency in the involvement of school nurses in students’ weight management education [8]. The National Association of School Nurses (NASN) recognizes the role of the school nurse in promoting the prevention of overweight and obesity and has developed a comprehensive toolkit with resources to assist nurses with the management of overweight and obese youth [9]. Despite existing resources, state-specific mandates to school nursing practices make a one-size-fits-all approach to providing educational opportunities and support for students with obesity difficult. School nurses who are introduced to a toolkit, like that of the NASN, are offered an identifiable and feasible role in that of childhood obesity prevention training [10].

One strategy that has been used since April 2000, when the Centers for Disease Control and Prevention released body mass index (BMI) screening tools for males and females 2-20 years old, is for schools to conduct their own BMI screenings among their student population. Consistent with early identification practice criteria, the Institute of Medicine and the American Academy of Pediatrics identified BMI screening as a practice that should be implemented by schools annually to help children and their parents understand and address healthy body weight [11-13]. Currently, 25 states across the United States mandate BMI screenings or weight-related assessments in their public schools in which only 44% of those states require parental notification [14]. The inconsistency in additional follow up by schools for parent inquiries may prevent affected children from receiving treatment. While school nurses serve as the liaison between the school and parents in communicating health concerns, they cite barriers to addressing obesity with their students and families for a variety of reasons, including lack of parental engagement, inadequate knowledge, lack of support, societal norms, and low perceived competency [7,15]. At the core of the NASN Framework for 21st Century School Nursing Practice [16] is student-centered health care in the context of the student’s family and school community. This underscores the need to identify the resources and tools school nurses would find most helpful in improving communication and engagement with families and addressing weight-related health with their students.

Although BMI screening results are useful for educating parents and encouraging follow up with a health care provider when necessary, this information can also be used to monitor aggregate changes in BMI year to year and evaluate obesity prevention programs in the school setting. School nurses should be considered leaders for implementing effective school-based programs, as their involvement has resulted in improvements in BMI percentile in several studies [6]. Therefore, additional training opportunities to do so creates benefits beyond that of the role of the school nurse and, instead, expands to benefit students and the larger community [16,17]. Furthermore, nurse self-efficacy in performing childhood obesity prevention practices significantly influences involvement [18], underscoring the importance of determining professional development opportunities, specifically those that overcome traditional barriers related to time and financial resources [19]. Pennsylvania (PA) is 1 out of 11 states requiring BMI screening and BMI result reporting to parents or guardians [14]. Therefore, this study, focusing on PA school nurse BMI screenings, will provide insight into the efficacy and follow through of the school’s role in childhood obesity prevention. We conducted a state-wide survey of school nurses in PA to understand current areas of care, find ways to address child health through school BMI screenings and follow up, and identify current educational gaps to assist school nurses with providing whole child care within the realm of weight management and education.

## Methods

### Design

This study included a cross-sectional Web-based survey delivered via email to 500 public school nurses in PA. Combined listservs for Penn State Health, PRO Wellness, and Penn*Link, an email listserv developed through the Pennsylvania Department of Education, were used to distribute the survey. All study and recruitment procedures were approved by the authors’ institutional review board.

### Procedure

School nurses were contacted in April 2016 to complete the survey through email invitation. The survey was open for 1 month and 2 reminders were sent to encourage participation. Inclusion criteria included school nurses in PA schools who were English-literate. Participants were prompted with consent language prior to entering the survey; thus, entering the survey implied consent to participate. The survey consisted of closed-ended, multiple choice, open-ended, and Likert-type scale questions and required 15 minutes to complete. Although survey respondents were anonymous, they were directed to a contact form on the Penn State PRO Wellness website to provide contact information for receipt of a gift card to compensate them for completing the survey.

### Instrumentation

The purpose of this study was to conduct a PA state-wide 29-item survey addressing school nursing barriers and practices, supplementing information already known in this area. The study team worked collaboratively with the Director of the Division of School Health at the Pennsylvania Department of Health to design the survey. Questions were based upon identified information gaps. The survey was researcher developed consisting of 3 broad categories of information. The categories included (1) demographic of the school; (2) primary areas of care; and (3) general development and methodology of school nurse practices. It aimed to understand current practices and resource preferences for addressing student health and discuss BMI screening results and professional development needs. Although the survey covered a range of topics, the focus was BMI screening and its related practice within the schools.

**Figure 1:**
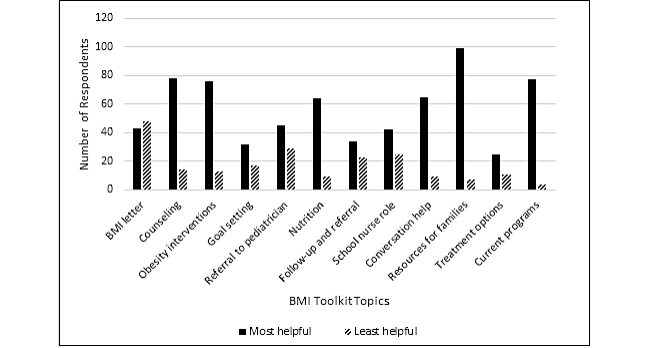
Body mass index (BMI) toolkit topics of interest.

Within the primary areas of care section of the survey, nurses were asked to share common areas of care provided to students on a daily basis and partnerships in completing mandated health screenings. Additionally, this section addressed BMI screenings by asking about BMI letter usage, inquiries, and follow up. Specific questions asked of school nurses included how school nurses notify parents of BMI screening results, whether or not they commonly receive inquiries, whether or not school nurses would benefit from a toolkit with resources to help communicate with parents and children about BMI screenings, topics of greatest and least interest to include in the toolkit, and format of toolkit information (Figure 1 and Table 1). Further questions asked about topics of interest for professional development, methods of delivery of educational opportunities, and areas of need for assistance.

**Table 1:** Keywords and survey responses.

Keyword	Original survey response
BMI^a^ letter	BMI screening letter
Counseling	Counseling and motivating overweight children and families
Obesity interventions	Current resources in obesity interventions (school-based)
Goal setting	Goal setting and follow up
Referral to pediatrician	Growth screening referral to pediatricians
Nutrition	Nutrition messaging
Follow up and referral	Protocol for ongoing follow up and referral
School nurse role	Role of the school nurse
Conversation help	How to talk about BMI with students and their parents
Resources for families	Tools for students and parents on nutrition and physical activity
Treatment options	Treatment options
Current programs	Wellness and weight management programs

^a^BMI: body mass index.

### Participants

Nurse participants (N=210), with a 42% (210/500) response rate, represented 208 school districts from urban (14/208, 6.7%), suburban (102/208, 49.0%), and rural (92/44.2%) locales within the state of PA. This is a representative sample of PA schools according to data obtained from “The Center for Rural Pennsylvania” [20], which indicates that 47.6% (238/500) of the districts classify as rural and 52.4% (262/500) as suburban or rural. On average, these districts employ between 4 and 19 full-time nurses depending on urban or rural classification, with a mean student population of 4170 students. Depending on the size of the school district, some school nurses serve all grade levels. All grade levels were represented in the sample, including pre-K (46/210, 21.9%), elementary (132/210, 62.9%), and secondary (94/210, 44.7%) levels.

### Data Analysis

Descriptive analysis was conducted using means and SDs. Chi-square test of independence was conducted to determine the relationship between BMI screening follow up and interest in BMI toolkit. The BMI screening follow up question “In addition to the BMI letter, how do you follow up with parents whose child screens overweight or obese?” was dichotomized, with responses of “phone call,” “email,” or “letter home” categorized as “yes” and “do not follow up” categorized as “no.”

## Results

### Body Mass Index Screening and Results

Participants were asked about their current process for notifying parents of BMI screening results. The majority (116/210, 55.2%) send a letter home in the mail, while others (62/210, 29.5%) send a letter home with students. A small number (8/210, 3.8%) said they did not notify parents altogether, and some notify parents electronically, either by email (8/210, 3.8%) or by posting in a parent portal (31/210, 14.8%).

More than one-third (75/210, 35.7%) of the nurses reported receiving BMI screening inquiries from parents; however, under half (35/75, 47%) of those respondents indicated they follow up with parents whose child screens overweight or obese. Overall, the vast majority (182/210, 86.7%) do not follow up with parents whose child screens overweight or obese. The majority of the nurses (150/210, 71.4%) responded they would benefit from a toolkit with resources to assist with communication with parents and children about BMI screenings. Of those that answered no (60/210, 28.6%), most (31/60, 51%) indicated that the parents are not interested in further discussion, do not take the screening seriously, or feel offended by the results. A significant association between respondent follow up and interest in a BMI toolkit was observed (*P*=.01).

For topics of inclusion in a BMI toolkit, participants were presented with a list of 12 options and asked to select 3 that would be most helpful to them and 3 that would be least helpful to them (Figure 1). The highest-ranking topics for a toolkit included tools for students and parents on nutrition and physical activity (99/210, 47.1%), counseling and motivating overweight children and families (78/210, 37.1%), wellness and weight management programs (77/210, 36.7%), and current resources in obesity interventions (76/210, 36.2%). School nurses prefer to receive this information via quick, one-page fact sheets (90/210, 42.9%), posters (85/210, 40.5%), or electronic or printable toolkit (81/210, 38.6%).

### Professional Development Needs

**Figure 2:**
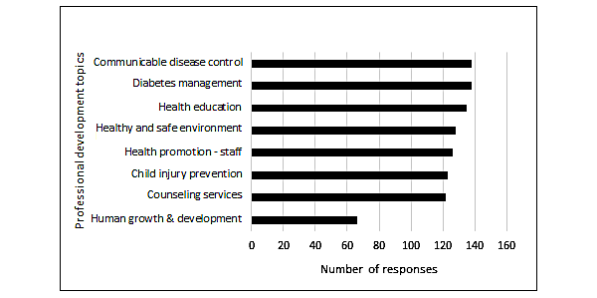
Areas of professional development of interest to school nurses.

To fully understand the breadth of a school nurse’s priorities and development, our survey expanded beyond the topic of BMI screening. The survey asked about which areas of professional development were of interest. High-ranking topics included diabetes management (138/210, 65.7%), communicable disease control (138/210, 65.7%), and health education (135/210, 64.3%). Additionally, 101 respondents provided write-in topics of interest, including mental health or drug abuse (22/101, 21.8%), nutrition or health and wellness (13/101, 12.9%), concussions or sports injuries (10/101, 9.9%), allergies or asthma (8/101, 7.9%), dermatology (6/101, 5.9%), and other miscellaneous topics such as community resources, legal issues, sexual health, and emergency care (Figure 2). For preferred method of information distribution, respondents selected self-paced Web-based course as their top choice (154/210, 73.3%), followed by recorded webinars (128/210, 61.0%), email newsletters (111/210, 52.9%), an annual health summit (69/210, 32.9%), and live webinars (44/210, 21.0%).

### Current Practices

Participants were presented with the question “Which of the following are the most common areas of care you provide to your students?” and were asked to respond to 7 different areas of care with either “very common,” “somewhat common,” or less common.” Respondents answered “very common” with the highest frequency to the following areas of care: acute illnesses (205/210, 97.6%), student injury (159/210, 75.7%), and chronic illnesses (132/210, 62.8%).

## Discussion

### Principal Findings

Overall, school nurses follow the PA mandate to complete BMI screenings with related communication to parents via a letter or email. However, though a large portion admitted to receiving inquiries from parents and students regarding BMI screening results, only half of those indicated they follow up on such inquiries. Also, overwhelmingly, the majority (182/210, 86.7%) did not follow up after initial notification of results to parents, indicating school nurses may require additional guidance for providing individualized support to students and parents about childhood overweight and obesity. Furthermore, the majority (150/210, 71.4%) indicated they could benefit from a BMI toolkit with resources to help communicate to parents about BMI screening results and to address weight-related concerns, and most (143/210, 68.1%) saw a need for more professional development opportunities that could aid them in serving as health champions and supporting whole child health in their districts. This study adds to the literature by examining educational needs and interests of PA school nurses, including specific topics of interest for a BMI toolkit and professional development.

Pennsylvania is 1 of only 25 states nationwide that require BMI screenings or weight-related assessments and only 1 of 11 states to require parent notification of results. Though most school nurses in the sample (201/210, 95.7%) responded favorably to communicating initial results to parents, an overwhelming majority (182/210, 86.7%) indicated that further follow up does not occur even if inquiries are received. This indicates a missed opportunity to provide guidance to families regarding lifestyle changes, improved nutrition, and other treatment options that could ultimately improve obesity rates. Though results from this study do not identify the reason for this lack of follow up, it has been well documented that school nurses face many barriers at individual, school, and societal levels that prevent them from feeling confident and able to address weight-related concerns with their students and families [7].

Many resources to support school nurses with weight-related health already exist, including the NASN obesity toolkit [9], though the specific screening requirements of PA schools may require the development of additional guidance to more adequately support nurses in PA and other states with similar mandates. Most (151/210, 71.9%) of the respondents indicated they would benefit from a BMI toolkit; furthermore, 92.8% (195/210) of those that currently follow up on BMI screening results expressed interest in a toolkit, and 67.6% (142/210) of participants who do not currently follow up on BMI screening results expressed interest in a toolkit, indicating that regardless of current follow-up practices, the majority saw benefit in a toolkit for assistance with parental communication. A significant portion of responses indicated they would not benefit from a BMI toolkit (109/210, 52%) citing lack of parental engagement as a reason. However, other studies have shown parents are primarily concerned with confidentiality and message content (ie, no weight-labeling) and do see benefit in receiving additional follow up to support healthy eating, physical activity, and overall lifestyle change in the home environment [21]. It is important to identify resources for school nurses that would help to improve self-efficacy, knowledge, and confidence when approaching parents with sensitive weight-related information. Better integration of public schools into children’s health care can result in systems of care that will more successfully manage and prevent obesity.

To our knowledge, there is little known regarding specific topics of interest that school nurses feel would assist them with improving weight-related communication with their students and families. To fill this gap, this study asked school nurses to identify 3 topics that would be most helpful for inclusion in a toolkit and 3 topics they feel would be least helpful for inclusion from a list of 12 topics. Based upon this information, school nurses prefer resources to counsel children and their families, educational materials, and information about current weight management programs and preventive interventions. Least helpful is information regarding the screening letter and growth screening referral, indicating that their interest in serving as facilitators of obesity prevention efforts may be greater than their interest in simply providing information. These results further help to identify materials nurses would most likely utilize and promote to beneficially supplement this BMI screening process.

In addition to targeted follow up with families of students who screen as overweight or obese, districts should acknowledge and support school nurses as leaders in overall school health by involving them in obesity prevention efforts and providing them with the resources, both time and financial, to take advantage of professional development opportunities to improve knowledge and self-efficacy. One survey conducted in a sample of 221 school nurses found that most (76%) supported the use of school health services for obesity prevention, but the same study further calls for time and support from the school for success [17]. Although it may seem counterintuitive to provide education and training to health professionals, literature suggests many health care providers, such as school nurses, are not comfortable providing these services [6,7]. A 2015 survey conducted by NASN with 8006 school nurses nationwide determined that “staff and student wellness” was one of the top 6 identified educational needs of school nurses (30.8% of survey) [22]. The survey in this study broke this down further into 8 categories to determine specific topic areas related to staff and student wellness. This data should be utilized by health educators and local agencies to provide meaningful continuing education opportunities that are of greatest interest to school nurses.

### Study Limitations

There are several limitations within the study design that should be addressed. For instance, the sample is limited to school nurses in the state of PA. As the data are derived from a single state, findings specific to BMI screenings and supporting resources may not be generalizable across other states; however, 25 states do require BMI screening or weight-related assessments. Furthermore, mandates on professional development requirements and required school screenings vary by state, so survey results from other populations may indicate different priorities. Questions posed in the survey were developed by the research team and Pennsylvania Department of Health after reviewing the more broadly disseminated NASN school nurse survey. Questions were created to supplement results already known regarding barriers and educational needs of school nurses, so this survey did not ask specific questions about barriers but rather inquired about specific topics of interest related to supplemental materials, resources, and trainings to help overcome known barriers. Furthermore, the reliability and validity of the measure is unknown.

### Conclusions

Despite juggling a host of student health services, school nurses fill a critical need in each district throughout the state in keeping children’s weight at a healthy level. It is the NASN’s position that the school nurse supports the physical, mental, emotional, and social health of students and their success in the learning process. Recognizing the important role school nurses play to monitor and promote children’s health, schools should involve them as key players in a role beyond anthropometric measurement when it comes to obesity prevention efforts. This goal might include involving them in school-based preventive programs, empowering them to lead initiatives that support whole child health and ensuring opportunities for professional development of interest to them. Nonetheless, the first step in facilitating obesity prevention methods within schools is to provide school nurses with meaningful tools that help facilitate conversations with parents, guardians and caregivers regarding their child’s weight status and health through a BMI screening toolkit. In PA schools, where BMI screening with parent notification is required, there is an opportunity to turn the added responsibility of this process into an opportunity to curb rising obesity rates.

## Abbreviations

BMI: body mass index
NASN: National Association of School Nurses
PA: Pennsylvania
